# Preventing Unequal Health Outcomes in COVID-19: A Systematic Review of Past Interventions

**DOI:** 10.1089/heq.2021.0016

**Published:** 2021-12-27

**Authors:** Beth E. Williams, Karli K. Kondo, Chelsea K. Ayers, Devan Kansagara, Sarah Young, Somnath Saha

**Affiliations:** ^1^ Primary Care, VA Portland Health Care System, Portland, Oregon, USA.; ^2^ Evidence Synthesis Program, VA Portland Health Care System, Portland, Oregon, USA.; ^3^ Research Integrity Office, Oregon Health and Science University, Portland, Oregon, USA.; ^4^ Department of Medical Informatics and Clinical Epidemiology, Oregon Health and Science University, Portland, Oregon, USA.; ^5^ Center to Improve Veteran Involvement in Care (CIVIC), VA Portland Health Care System, Portland, Oregon, USA.; ^6^ Division of General Internal Medicine, Oregon Health and Science University, Portland, Oregon, USA.

**Keywords:** COVID-19, health disparities, health inequities, systematic review

## Abstract

**Background:** We sought to identify interventions that reduced disparities in health outcomes in infectious disease outbreaks or natural disasters in the United States to understand whether these interventions could reduce health disparities in the current COVID-19 pandemic.

**Methods:** We searched MEDLINE and other databases to May 2020 to find studies that examined interventions to mitigate health inequalities in previous infectious disease pandemics or disasters. We assessed study quality using the Newcastle–Ottawa Scale and the Critical Appraisal Skills Program (CASP) Checklist for Qualitative Studies.

**Results:** We included 14 articles (12 studies) and 5 Centers for Disease Control (CDC) stakeholder meeting articles on pandemic influenza preparedness in marginalized populations. Studies called for intervention and engagement before pandemic or disaster onset. Several studies included interventions that could be adapted to COVID-19, including harnessing technology to reach disadvantaged populations, partnering with trusted community liaisons to deliver important messaging around disease mitigation, and using culturally specific communication methods and messages to best reach marginalized groups.

**Discussion:** To our knowledge this is the first systematic review to examine interventions to mitigate health inequities during an infectious disease pandemic. However, given that we identified very few disparities-focused infectious disease intervention studies, we also included studies from the disaster response literature, which may not be as generalizable to the current context of COVID-19. Overall, community outreach and tailored communication are essential in disease mitigation. More research is needed to evaluate systemic interventions that target the distal determinants of poor health outcomes among marginalized populations during pandemics and natural disasters.

## Background

Since the first cases of COVID-19 were detected in the United States in early 2020, the effect of the disease has varied greatly between and across socioeconomic classes and racial/ethnic groups. Recent studies have shown poorer health outcomes for those of lower socioeconomic status (SES) and racial/ethnic minorities and higher rates of hospitalization among African Americans (AAs)/Blacks and Latinos.^1,2^ Similar disparities in health outcomes have been observed in past infectious disease outbreaks.

In the 2009 H1N1 pandemic, AA/Blacks and Latinos in Illinois had higher rates of hospitalization and mortality.^3^ Patients of low SES in New York City were also found to have higher odds of hospitalization.^4^ While U.S. researchers have yet to assess the long-term impact of COVID-19 on disadvantaged populations, it is likely the United States will be feeling the ripple effects of these unequal health outcomes for years. For this reason, research on the root causes and potential mitigating strategies of these disparities is crucial.

We define health disparities as differences in health outcomes or health care use between socially distinct marginalized and less marginalized populations.^5^ In the current review, our approach is guided by a framework initially defined by Kilbourne^5^ and later refined by Saha^6^ and Thomas.^7^ This framework describes three phases of health inequality research, each informing the subsequent phase that include: detecting disparities, understanding disparities, and reducing disparities through targeted interventions.

This review focuses on the intervention phase. We base analysis of our findings on the foundational work of Quinn and Kumar, who offer a model for considering the potential causes of epidemic influenza based on measures of exposure, susceptibility, and access to care. They apply their model to data collected in 2009–2010 during the H1N1 pandemic.^8,9^

The model includes both distal or upstream (e.g., governmental policies and social determinants such as SES) and proximal or downstream (e.g., vaccination and handwashing) causes of disparate health outcomes that we use in this study to contextualize and categorize interventions. Through systematic review, we sought to identify interventions that have effectively reduced disparities in health outcomes in infectious disease outbreaks or natural disasters in the United States and understand whether such interventions could be applied to reduce health disparities in the current COVID-19 pandemic.

## Methods

This study is part of a larger systematic review commissioned by the Veterans Health Administration (VHA) that examined the mediating factors contributing to health-related inequalities in previous pandemics and the interventions developed to address them.

The protocol, which follows PRISMA guidelines,^10^ was registered to PROSPERO (CRD42020187078) before study initiation.

### Data sources and searches

We searched MEDLINE ALL, PsycINFO, Cochrane Database of Systematic Reviews, and Cochrane Central Register of Controlled Trials from database inception through May 20, 2020. Searches included controlled vocabulary terms (e.g., MeSH), along with free-text words, related to previous epidemics, pandemics, disasters, and disparities. We reviewed the bibliographies of relevant articles and contacted experts to identify additional studies. Search strategies were developed in consultation with a research librarian (Supplementary Appendix SA1).

We further refined search results by performing keyword searches in EndNote (X9.3.3) to exclude articles that are not studies (i.e., errata, comments, replies, proposals), basic science studies, animal studies, studies that were not of infectious disease pandemics, epidemics, or disasters relevant to the United States, and studies of non-U.S. state or territory populations. Titles and abstracts excluded through keyword search were confirmed by another investigator.

### Study selection

Eligible studies included adult U.S. populations and examined interventions to mitigate health inequalities in a previous disaster or infectious disease epidemic or pandemic by race/ethnicity, SES, disability, or geographic location (Supplementary Appendices SA2 and SA3).

We anticipated that we would find relatively few intervention studies, so we also included qualitative studies and program evaluation studies that described “lessons learned” about intervention preparation and implementation, since this information could be used to inform the design of future interventions. We also included articles describing recommendations for intervention development from stakeholder meetings. Studies were independently reviewed by at least two reviewers. Discordant results were resolved through consensus or a third reviewer.

### Data abstraction and quality assessment

From each included study, we abstracted sample size, setting, population characteristics, inclusion and exclusion criteria, intervention description, and findings. Data were abstracted by one investigator and confirmed by a second. Two reviewers independently assessed study risk of bias using modified versions of the Newcastle–Ottawa Scale for observational studies, the Critical Appraisal Skills Program (CASP) Qualitative Checklist for qualitative studies (Supplementary Appendix SA4).^11,12^ We did not assess the risk of bias of articles that were not research studies. Disagreements were resolved by consensus or a third reviewer.

### Data synthesis

The studies and expert panel reports included in this review varied greatly in objective, design, and methodology, which precluded quantitative analysis of combined results across studies. As a result, key findings were abstracted from each study, grouped by study type, and reported qualitatively in the following text and attached tables.

## Results

**FIG. 1.**
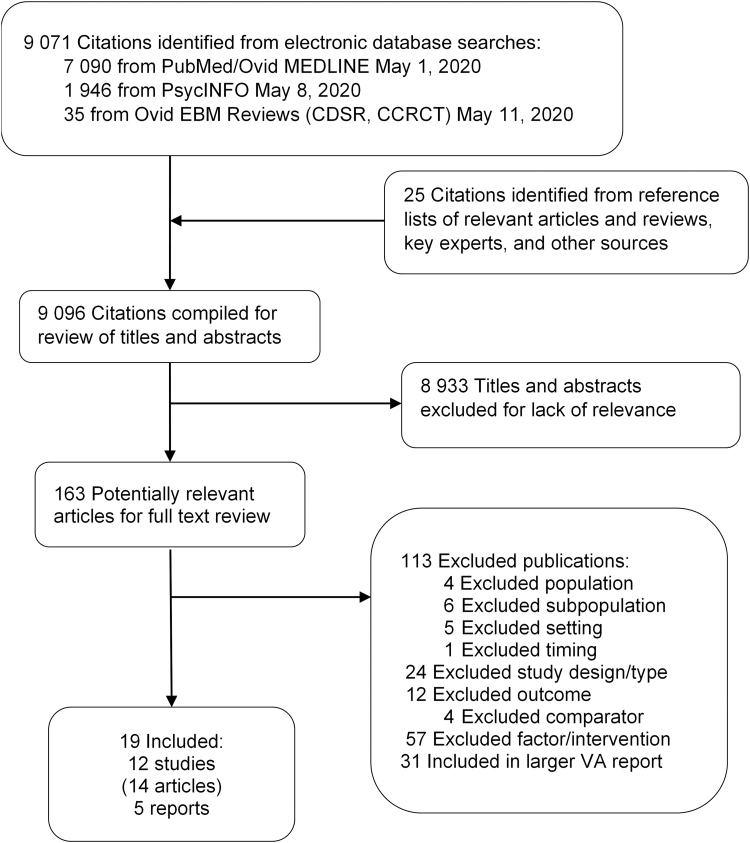
Literature flow chart.

We reviewed 9098 titles and abstracts, and 163 full-text articles; 12 studies (14 articles) met criteria for inclusion (Fig. 1).

There are four studies (five publications) with individual-focused interventions,^13–17^ and eight studies (nine publications) with system-focused interventions.^18–26^ Of these, only one^27^ reported intervention effectiveness outcomes, while the majority described acceptability and feasibility of the studied interventions. We found only one randomized control trial (RCT) and one longitudinal cohort study. The remainder of the articles reviewed were program evaluations or studies to inform future interventions with resulting recommendations. Tables 1 and 2 provide study details.

**Table 1.** Articles by Population

Article Author, year n* participants Setting Dates Study design Study timing *Focus	Demographics % female Age (SD) Race/Ethnicity Education Unemployed Other	AA/Black	Latino	Asian and/or Pacific Islander	American Indian or Alaska Native	Limited English proficient	Low socioeconomic status	Rural	Disability	Applicability	Study quality ratings and concerns
Andrulis, 2011^33^ (1) *n*=17 California statewide +4 regions (Central Coast, Bay Area, Central Valley, and Los Angeles) June and August 2008 Qualitative: key informant interviews *Disaster planning*	6 Nonprofit agencies; 3 County; PH Depts; 3 CBOs; 2 Local EMR orgs; 2 State agencies; 1 Academic	✓	✓	✓	✓	✓	✓			Fair	Generally good, lacks some methods reporting.
Aten, 2010^19^ (2) Aten, 2011^20^ (3) *n*=41 Southern Mississippi (Hancock, Harrison, and Forrest Counties) ∼1 year after Hurricane Katrina Qualitative: semistructured interviews with pastors of AA churches *Post disaster mental health disparities*	7% female Age: 51.2 (10.81) 1 Catholic; 2 African Methodist Episcopal; 38 Missionary Ministry experience: 16.91 (10.41)	✓								Fair	No issues. Good qualitative methods described.
Bouye, 2009^28^ (4) *n*=26 CDC stakeholder meeting May 1–2, 2008 *Influenza pandemic: low-SES, public housing residents, single-parent families*	Federal, State, and Local HUD Depts.; State and Local agencies; CBOs and FBOs; Academics; Community Members						✓			Good	N/A: CDC stakeholders
Eisenman, 2009^27^ (5) and Glik, 2014^14^ (6) *n*=187 Los Angeles County 2006–2007 Randomized longitudinal cohort *Disaster preparedness intervention*	I vs. C % female: 66.67% vs. 68% Age: 37.08 vs. 36.97 ≤HS: 77% vs. 75% HS+: 23% vs. 25% Below FPL: 66.67% vs. 71% Not working: 24.14% vs. 29%		✓			✓				Fair	Fair quality
Goodman, 2009^21^ (7) *n*=6 New Orleans, LA post-Hurricane Katrina *Culturally competent disaster response mental health students*	100% female Age: 31 1 Haitian American 1 Indian American 4 European Americans	✓								Fair	N/A: program evaluation
Hutchins, 2009^29^ (8) *n*=NR CDC stakeholder meeting May 1–2, 2008 *Influenza pandemic: racial and ethnic minorities*	State and local PH officials; health care providers, state and local EMR professionals, Academics, CBOs, FBOs, advocacy organizations, racial and ethnic minorities	✓	✓	✓	✓	✓				Good	N/A: CDC stakeholder
McCabe, 2013^26^ (9) *n*=178 Maryland Date: NR Program evaluation *Community-level disaster preparedness*	Faith-based Participants: 73% female AA/Black: 31% Latino: 1% Biracial: 2fx%							✓		Fair	N/A: program evaluation
Nassar, 2014^15^ (10) *n*=50 Washington, DC 2010 Feasibility RCT *Monitoring H1N1 symptoms*	100% women I vs. C Age: 24.1 (6.3) vs. 23.5 (5.2) AA/Black: 87% vs. 84.6% Medicaid eligible: 87% vs. 80.7%						✓			Fair	Poor quality, unblinded, small feasibility trial
Obaid, 2017 (11) *n*=667 participants Rural Nebraska 2010–2013 Program evaluation *Rural infectious disease disaster preparedness*	83 agencies across 3 medical response systems/coalitions Also: EMS, fire, emergency management, county officials, health care and public health staff							✓		Fair	N/A: program evaluation
Person, 2004^23^ (12) *n*=70+ people, 50 agencies/orgs April 2003 NCID/CDC response Focus groups (11) *SARS-related stigma*	Chamber of commerce, trade associations, school officials, public health, mental health professionals, academics			✓						Good	N/A: NCID/CDC
Price, 2013^16^ (13) *n*=1180 2 South Carolina counties (Galveston and Chambers) ∼1 year post-Hurricane Ike Pre/post feasibility study, qualitative follow-up *Post-disaster mental health intervention*	50.7% female Age: 47 (17) AA/Black: 13.7% Latino: 6.3% White: 80% HS: 21.4% Some college: 36.1% <$40K: 21.4%	✓	✓							Fair	—
Rosenbaum, 2018^17^ (14) *n*=22 (completed training) Oceana County, MI June and October 2016 Program evaluation *Disaster preparedness training for MSFW*	NR		✓			✓				Fair	N/A: program evaluation
Steege, 2009^30^ (15) Briefing report from National Farmworker Health Conference and Western Migrant Stream Forum organizers May 2008 *Influenza pandemic: farmworkers*	NR		✓			✓				Good	N/A: report
Truman, 2009^31^ (16) CDC stakeholder meeting May 1–2, 2008 *Influenza pandemic: immigrants and refugees*	Public health scientists Service program managers	✓	✓	✓		✓				Good	N/A: CDC
Vaughan, 2009^32^ (17) CDC stakeholder meeting May 1–2, 2008 *Influenza pandemic: risk communication and vulnerable populations*	Public health experts Program managers	✓	✓	✓	✓	✓	✓		✓	Good	N/A: CDC
Wyte-Lake, 2014^24^ (18) *n*=7 Single urban VHA HBPC program Qualitative: semistructured interviews *Disaster preparedness*	Associated chief of staff Program manager HBPC practitioners (nursing, OT, social work, psychology)								✓	Fair	Small study, poorly reported
Wyte-Lake, 2019^25^ (19) *n*=754 patients 10 VHA HBPC sites in 8 states April–October 2017 Cross-sectional survey evaluating a patient assessment tool *Disaster preparedness*	16% high risk; 44% medium risk; 40% low risk 25% on oxygen 30% chair-bound 55% assistive device 33% cognitive impairment 20% communication limitation								✓	Poor	No control for confounders, methods poorly reported

AA, African American; AI/AN, American Indian/Alaska Native; C, control; CBO, community-based organization; CDC, Centers for Disease Control; EMR, electronic medical record; EMS, emergency medical services; FBO, faith-based organization; FPL, federal poverty limit; HS, high school; HBPC, home-based primary care; HUD, U.S. Department of Housing and Urban Development; I, intervention; LA, Los Angeles; LEP, limited English proficiency; MSFW, migrant and seasonal farmworkers; N/A, not applicable; NCID, National Center for Infectious Disease; NR, not reported; OT, occupational therapy; PH, public health; PI, Pacific Islander; RCT, randomized controlled trial; SARS, severe acute respiratory syndrome; SD, standard deviation; SES, socioeconomic status; US, United States; VHA, Veterans Health Administration.

**Table 2.** Relevant Findings from Infectious Disease and Emergency Response Literature

Author, year n participants Focus Population	Intervention or program description; comparator	Lessons learned Relevance for COVID-19
Individual-focused interventions and programs		
Eisenman, 2009^27^ and Glik, 2014^14^ *n*=187 *Disaster preparedness intervention* • Latino (English and LEP)	Intervention: Emergency preparedness program (two groups: high-intensity [*platicas*=small group discussions with community health worker]; and low-intensity [culturally appropriate mailers]) Comparator: culturally appropriate mailing	• Importance of working with trusted community-based organizations to help translate disaster preparedness messages for disadvantaged households • More focused community-based outreach than current standard practice is needed; reliance on mass-media campaigns to disseminate messages may be unreliable *Delivery of information and services related to COVID-19 to vulnerable populations will likely be more effective if delivered via trusted community intermediaries and targeted community outreach efforts rather than via print media alone.*
Goodman, 2009^21^ *n*=6 *Culturally competent disaster response mental health students* • AA/Black	Cultural competence program; 8-day outreach experience providing disaster response counseling services, accompanied by journal and processing with peers and faculty supervisor	Results highlighted: • Developing cultural competence of disaster response counselors can be achieved through outreach experience with processing using a critical consciousness lens. *A critical consciousness-based approach could be useful in training counselors to provide culturally competent counseling to marginalized individuals who experience trauma related to COVID-19.*
Nassar, 2014^15^ *n*=50 *Monitoring H1N1 symptoms* • Low SES	Intervention: daily automated calls re: flu symptoms. If yes, they were transferred to a nurse midwife for triage and next-day visit. If they did not respond to automatic calls for 3 days, they were called. Comparator: health education	• An automated system for triage of symptoms and referral to care could help reach disadvantaged populations affected by COVID-19
Price, 2013^16^ *n*=1180 *Post-disaster mental health intervention* • AA/Black • Latino	Intervention: brief, web-based disaster mental health intervention carried out 1 year after hurricane Ike. Modules included depression, PTSD, generalized anxiety disorder, panic disorder, alcohol abuse, marijuana abuse, and cigarette smoking. Engagement was assessed based on three types of attrition.	• Rates of attrition for use of a web-based mental health intervention did not differ between AAs, Latinos and Whites. *Web-based interventions related to COVID-19 could be useful to reach AAs, Latinos, and Whites at similar rates.*
Rosenbaum, 2018^17^ *n*=22 *Disaster preparedness training for MSFW* • Latino (LEP)	Two disaster preparedness workshops were conducted with migrant and seasonal farm workers using the Community Emergency Response Team curriculum that includes basic disaster response skills such as fire safety, light search and rescue, team organization, incident command, and disaster medical operations.	• Participants improved emergency preparedness and first aid, CPR, and AED competencies through workshop participation • Partnerships with the university and the relevant local stakeholders were important to project planning and implementation. • Needs of participants such as work/school schedules, transportation, and childcare needs must be considered • Bilingual trainer and training materials are important • Curriculum needs to be culturally relevant *In person COVID-19-related training of migrant and seasonal farm workers is important, needs to include culturally appropriate trainers and materials, and needs to accommodate work and personal needs of participants to allow for participation.*
System-focused interventions and programs		
Andrulis, 2011^33^ *n*=17 *Disaster planning* • AA/Black • Latino (English and LEP) • Asian/PI • AI/AN	Through literature review, environmental scan of organizational websites, and 17 key informant interviews with public health and emergency management personnel, researchers identified barriers and disaster preparedness needs of racially/ethnically diverse communities.	Results highlighted: • Barriers to preparedness include socioeconomic factors, trust in perceived fairness of government, cultural and linguistic factors, lack of funding for diversity initiatives, limited knowledge about and collaboration with diverse communities • Program and policy priorities: enhance collaboration; increase flexibility for program development and allocation of funds; improving organizational capacity • Intervention priorities: engage diverse communities; mitigate stigma and fear; build cultural competence; coordinate information and resources *Many of the barriers to reaching racially and ethnically diverse communities that were identified in this study also apply to COVID-19. Outreach efforts must employ cultural competence, enhance collaboration, and leverage resources to enhance organization capacity.*
Aten, 2010^19^ Aten, 2011^20^ *n*=41 *Post disaster mental health disparities* • AA/Black	Pastors of churches in South Mississippi affected by hurricane Katrina participated in semistructured interviews 1 year after the storm. Results were synthesized to provide recommendations for fostering collaboration between AA/Black religious leaders and mental health professionals toward better serving minority communities.	Recommendations: • Establish working relationships before disasters • Empower AA churches through participation, both empowering AA faith communities to utilize spiritual resources, but also providing leadership opportunities for pastors and congregation members • Utilize AA churches for community-based services: bringing services to the community can increase access and utilization. *AA pastors and churches could be an essential ally when considering interventions to mitigate disproportionate effects of COVID-19 on AA communities.*
McCabe, 2013^26^ *n*=178 *Community-level disaster preparedness* • Rural	Disaster/emergency preparedness intervention with 1-day didactic session and 2-day technical workshop focused around disaster preparedness and partnerships between faith-based organizations and local health departments:	*Providing training for FBOs through established partnerships could be 1 method of reaching marginalized communities with information and resources related to COVID-19.*
Obaid, 2017^22^ *n*=667 participants *Rural infectious disease disaster preparedness* • Rural	Functional infectious disease disaster response exercises, developed by Center for Preparedness Education at the University of Nebraska Medical Center	• Disaster response exercises are feasible as one way of assessing preparedness of medical and public health systems. *Provides a model for assessing preparedness of medical and public health systems before onset of an infectious disease disaster such as COVID-19. Could be applied in anticipation of future disease outbreaks.*
Person, 2004^23^ *n*=70+ people, 50 agencies/orgs *SARS-related stigma* • Asian	NICD/CDC SARS Community Outreach Team Activities: (1) advised other SARS emergency response teams on how to minimize the risk of stigmatizing groups in their own communications by focusing messages on the virus and the relevant behavioral risk factors; (2) assisted with developing culturally tailored health education materials; and (3) conducted community visits, panel discussions, and media interviews to positively influence negative behaviors occurring in communities	• The need to develop simple, tailored infectious disease prevention messages and materials in various Asian languages • Disseminate information through multiple and culturally appropriate channels, including (but not limited to) community visits and town hall meetings
Wyte-Lake, 2014^24^ *n*=7 *Disaster preparedness* • Disability	Seven interviews were conducted with HBPC providers to explore issues regarding emergency management planning for homebound patients.	• HBPC needs to increase disaster preparedness include: (1) training to focus on better strategies to get patients to participate, (2) more consistent time spent on patient education, (3) formalizing the initial assessment to actually evaluate how prepared patients are, and (4) having emergency preparedness be formally addressed on a more consistent basis, *HBPC providers are uniquely positioned to provide education and intervention around disaster preparedness to vulnerable patients. This could include provision of education about COVID-19. Efforts should be made to standardize COVID-19 preparedness assessment among HBPC providers.*
Wyte-Lake, 2019^37^ *n*=754 patients *Disaster preparedness* • Disability	Evaluation of the HBPC Patient Assessment Tool—tool to assess disaster preparedness among homebound vets. The rates at which education was provided on various items was assessed based on patient risk categorization to observe patterns in how providers communicated this information.	• Home health agencies can play an important role in educating home-bound adults about disaster preparedness. These results indicate that providers are giving basic education on disaster preparedness to their most vulnerable patients, but opportunities exist for improvement *Home health agencies may be an important partner in disseminating COVID-related education to vulnerable home-bound adults.*

AED, automated external defibrillator; CI, confidence interval; CPR, cardiopulmonary resuscitation; LA, Los Angeles; OR, odds ratio; PTSD, posttraumatic stress disorder.

We additionally included five articles presenting expert recommendations from Centers for Disease Control (CDC) key stakeholder meetings on pandemic influenza preparedness in disadvantaged populations convened in 2008.^28–32^ Tables 1 and 3 provide details.

**Table 3.** Relevant Findings from Centers for Disease Control Expert Panel Meetings on Impact of Influenza in Vulnerable Populations

Author, year n participants Topic	General recommendations/findings	Strategic partnership recommendations
Bouye, 2009^28^ *n*=26 *Influenza pandemic: low-SES, public housing residents, single-parent families*	• Use culturally specific communication to impart messages related to vaccines and hygiene • Engage strategic partnerships to relay public health messaging • Create defined school policies, provide childcare vouchers, and stockpile supplies at churches and community centers • Government support for workers—aid packages and wage freezes. • Workplace flexibility and competitive compensation • Preparation for delivery of goods and services through home delivery, mobile clinics • Education around signs and symptoms of pandemic influenza	Engage faith-based organizations, CBOs, and neighborhood planning units
Hutchins, 2009^29^ *Influenza pandemic: racial and ethnic minorities*	• Participatory approach to planning and preparedness process, engaging racial and ethnic minorities in every step of the process, and allotting funding to do so. • Social safety net policies and procedures are needed to meet survival needs, including access to clean water, sufficient food, shelter, and utilities. • Education materials that are culturally appropriate and adapted to low-literacy populations • Educating early about use of PPE • Systems for equitable access to scarce resources, including antiviral medications and vaccines	
Steege, 2009^30^ *Influenza pandemic: farmworkers*	• Collaboration between federal, state and local public and animal health and agriculture authorities • Seasonal influenza vaccination • Training on reduction of risk of infection • Sufficient PPE • Sanitary facilities • Surveillance and early detection of disease in workers and animals • Linguistically and culturally appropriate information about vaccination • Emergency messaging through multiple media • Two-way information system to reach farm worker in remote encampments	Federal, state, and local public and animal health and agriculture authorities should collaborate with farm employers, farmworker health and social service organizations, agricultural extension agencies, and farmworker advocacy groups
Truman, 2009^31^ *Influenza pandemic: immigrants and refugees*	• Provision of information regarding importance of staying at home while ill • Liberal workplace leave and teleworking policies • Engage faith-based and community organizations in how to best work with families to avoid social stigma in case of needed quarantine • Work with providers of services to immigrants and refugees on appropriate use, distribution, and barriers to use of PPE • Consider means to provide vulnerable children with lunch when schools are closed • Vaccine distribution in states where immigrants/refugees are overrepresented and along the southern border • Locate vaccination spots in easily accessible locations and provide vaccination without asking about immigration status	Faith-based organizations, community organizations and leaders, service providers
Vaughan, 2009^32^ *Influenza pandemic: risk communication and vulnerable populations*	Communication strategy for during pandemic: • Phased—public health information should track with phase of pandemic (mild, moderate, severe outbreak) • Communications should be situation-specific; schools and childcare centers, workplaces, and public and social gatherings, all require different communication strategies • Use of multiple communication strategies • Focus on understanding factors that affect subgroup differences in response to infectious disease and to develop and aggregate communication strategies that strengthen rather than diminish • All messages, materials, and documents should be culturally sensitive, match the language proficiency of targeted individuals, and be responsive to the changing conditions and needs of the audience as the crisis unfolds. • PPE: affordability, accessibility, availability, and appropriateness are real considerations for vulnerable populations. Equally important are language considerations and ensuring that instructions on how and when to use PPE (e.g., masks, gloves) are clear and workable.	Partnerships with community organizations, faith-based organizations, and trusted communication intermediaries

PPE, personal protective equipment.

### Individual-focused interventions and programs

Four studies (five articles) featured interventions focused at the individual level. One longitudinal cohort study aimed to improve emergency preparedness among a low-income Latino community. Participants (*n*=231) were randomized to receive either culturally tailored information sent by mail or to participate in discussion groups led by a community health worker. The discussion groups arm reported better disaster preparation postintervention than the mailer-only group.^27^ In a subsequent publication, the authors attributed the success of the emergency preparedness discussion groups to the use of targeted outreach provided by community-based organizations and the use of clear, consistent, culturally appropriate messaging.^14^

One small (*n*=50) RCT evaluated the feasibility of an automated call-monitoring system to detect H1N1 symptoms among low-income pregnant women. In addition to an individualized health education session, the intervention group received daily calls with prompts for yes or no responses for H1N1 symptoms. Participants who responded “yes” were transferred to a nurse midwife, and a same-day appointment was scheduled. Findings indicated that the intervention was feasible. There was no difference between groups in prenatal care visit attendance. Nearly all (93.3%) women in the intervention group recommended a similar system in future health crises.^15^

Another article describing a program evaluation analyzed differences in completion rates by race/ethnicity of a post-disaster web-based mental health intervention. The intervention included a baseline interview and mental health screen, after which participants (*n*=1249) were directed to relevant online modules addressing posttraumatic stress disorder, depression, generalized anxiety disorder, panic disorder, marijuana abuse, alcohol abuse, and smoking. The evaluation found that rates of access, use, and completion of the intervention did not differ between AA/Blacks, Latinos, and Whites.^16^

Finally, a program evaluation examined an intervention to train migrant and seasonal farmworkers in disaster preparedness through two workshops utilizing the Community Emergency Response Team curriculum.^17^ Results showed that the intervention was feasible and the workshops were highly rated by participants (*n*=22), but challenges existed with reaching recruitment goals. Evaluation indicated a need for partnering with stakeholders and accounting for participants' work schedules and language needs in planning the training.

### System-focused interventions and programs

Eight studies (nine publications) focused on system-level interventions to improve emergency preparedness and response. These are categorized below as either program evaluations^21,22,26^ or studies to inform future interventions.^19,20,23–25,33^

#### Program evaluations

A qualitative evaluation of a program that aimed to increase efficacy of counseling provided to ethnically and culturally diverse populations after a natural disaster used a critical consciousness approach to train psychology graduate students (*n*=6).^21^ The authors found that the experience increased participants' cultural competence and social justice-oriented perspective. While the effects on the care the students provided were not directly evaluated, this training potentially increased their efficacy at serving a majority AA/Black community.

McCabe et al.^26^ described a disaster/emergency preparedness training for lay communities in a rural region of Maryland with the goal of enhancing capacity of rural emergency response through a system-based partnership between faith-based organizations, local health departments, and academic institutions. A post-intervention assessment demonstrated an increased understanding among participants (*n*=178) of community disaster and mental health plans and increased self-efficacy to execute these plans, as well as potential for scaleup.

Another study described a program evaluation of a cross-agency rural health system response simulation exercise for communities in rural Nebraska.^22^ The evaluation utilized functional exercises: 3-hour disaster simulation exercises followed by a 3-hour regional debriefing conference. The exercises were assessed based on participant feedback, agency debriefing, and regional after-action reports with a focus on assessing command-level decision making and operations at multiple agencies.

The exercise highlighted system weaknesses that included difficulties with backup communication; lack of knowledge around how to request additional medical staff and assets or make patient transfer requests; the need to develop coordinated public messaging; and deficits in intra-agency coordination, for example not initiating a report of a notifiable disease. Overall, the program demonstrated the utility of functional exercises for testing regional disaster response coordination.

#### Studies to inform future interventions

A qualitative study consisting of 41 interviews with pastors of AA/Black churches in Southern Mississippi recommended that mental health professionals build relationships with churches before disasters and work to engage and empower pastors and congregants, building trust. Through these partnerships, mental health services could potentially be brought to the communities during disasters, increasing access.^19,20^

In another study, researchers completed a situational analysis of emergency preparedness of diverse communities in California. The study included a literature review, an environmental scan of organizational websites, and key informant interviews (*n*=17). It showed that there is a need to better engage diverse communities in all stages of disaster preparedness and response, mitigate stigma and fear, build cultural competence, and better coordinate information and resources when planning for community disaster preparedness.^33^

Person et al. (2004) performed a situational analysis of stigmatization of Asian communities in the United States during the SARS pandemic in 2003.^23^ The analysis included group discussions with key informants (*n*=70) from 50 different organizations, monitoring of the CDC public response service hotline, and a scan of Asian language information services. Results demonstrated significant stigmatization and misinformation related to SARS. Important recommendations gleaned from these findings included the need to develop simple, tailored SARS prevention messages and materials in various Asian languages and to disseminate SARS information through multiple culturally appropriate channels, including (but not limited to) community visits and town hall meetings.

Wyte-Lake et al.^24^ conducted seven interviews with Home-based Primary Care (HBPC) providers to explore issues regarding emergency management planning for homebound Veterans. The qualitative analysis showed that a lack of standardized policies and procedures and unclear designation of provider responsibility resulted in inconsistent preparedness among HBPC patients. Recommendations included better training of providers to assist their patients in disaster preparedness and formalization of the preparedness evaluation and intervention process.

One additional study evaluated the use of a disaster preparedness assessment tool among homebound Veterans enrolled in HBPC.^25^ The assessment tool was deployed with patients at 10 HBPC sites in 8 states (*n*=754) over a 3-week period. Results showed that, in general, providers were teaching basic skills of disaster preparedness to their most vulnerable patients. Evacuation planning was the most commonly covered topic, and Veterans in the high- or medium-risk categories were more likely to receive preparedness information than those in the low-risk category.

### Expert panel articles

Five articles presented expert recommendations resulting from CDC key stakeholder meetings on pandemic influenza preparedness in marginalized populations. These meetings were held in 2008, with reports published in an American Journal of Public Health supplement in 2009.^29–32,34^ Experts were convened around the following marginalized groups: publicly housed, single-parent families, low-income families, racial and ethnic minorities, migrant farm workers, and immigrants and refugees. One additional article addressed communication strategies to marginalized populations in general (Table 3).

In Bouye et al.,^28^ the expert group highlighted the ways in which poverty made those who are publicly housed, some single-parent households, or low wage earners more susceptible to a pandemic outbreak. Early implementation of community mitigation strategies was considered paramount to halt the spread of infection. The panel recommended engaging community liaisons in devising education campaigns as being essential to their success. Many of the strategies discussed hinged on preparedness planning before the onset of a pandemic to build the needed community and policy infrastructure to enable mitigation strategies (e.g., flexible work policies, home delivery services).

Another expert group meeting report^29^ looked at the disproportionate effect of influenza on racial and ethnic minorities. Similar to Bouye et al.,^28^ they proposed possible solutions that hinge on tailored educational strategies and strengthening of public health infrastructure. The group emphasized the importance of involving racial and ethnic minorities at all stages of planning and prevention to address the socioeconomic, cultural, linguistic, and educational barriers that may prevent community mitigation and vaccine delivery.

Steege et al.^30^ argued for the need for a distinct approach to protecting migrant farm workers against pandemic influenza. Migrant workers constitute a unique group in that they risk contracting disease due to their exposure to animals. Furthermore, working conditions and cultural or linguistic barriers may make an influenza outbreak more likely among this marginalized group.

Mitigation strategies focused on delivery of timely prevention and treatment of influenza. Two other recommended strategies included the use of mobile clinics and lay community health workers. Given that some farm workers who are undocumented may delay seeking care due to fear of legal repercussions, establishing trusting relationships with service providers to enable care delivery was considered important.

Immigrants and refugees share important risk factors with the aforementioned marginalized groups.^31^ This group may be at higher risk for pandemic influenza due to factors, including higher rates of chronic conditions, lower seasonal influenza vaccine rates, and linguistic or cultural barriers. In the case of undocumented immigrants, they may be reluctant to seek care due to fear of detention or deportation, similar to migrant farm workers.

Steege et al. proposed a multilevel approach to risk mitigation, with strategies that are household-focused (e.g., remaining home while ill), provider-supervised (e.g., vaccine provision), and agency-driven (e.g., effective communication). This expert group also emphasized involvement of the target group in all preparedness planning and communication.

One final article^32^ focused on general communication strategies to marginalized groups during an influenza pandemic. They recommended that communication strategies be tailored to the phase of the pandemic and that they be situation specific. For example, schools, workplaces, and public gatherings all require different communication strategies. A participatory approach was considered important to the development of communication materials, with dissemination best done in partnership with community organizations, faith-based organizations, or other trusted intermediaries (see Table 3 for details).

## Discussion

Through a review of the available literature, we sought to identify interventions that have been used to reduce health inequalities in infectious disease transmission or health outcomes in disasters, infectious disease epidemics, or pandemics in the United States with the ultimate goal of applying lessons learned from these efforts to the current COVID-19 pandemic. Considering the breadth of the topic, relatively few studies qualified for inclusion.

Of the 12 studies that described interventions or intervention components, only one study^13^ examined effectiveness outcomes, while the majority described acceptability and feasibility of the studied interventions. We identified only one RCT and one longitudinal cohort study. Additionally, five articles offered expert recommendations for how to address health disparities among marginalized communities in the setting of an influenza pandemic.

To our knowledge, this is the first review of studies aimed at mitigating health disparities in infectious disease epidemics or natural disasters. Much of the literature included in this review draws on prior research on mediating factors that contribute to health inequities. This includes the framework of Quinn and Kumar that points to proximal and distal determinants of disease burden with the ultimate goal of identifying potential points of policy and programmatic intervention.

While the recommendations generated from CDC stakeholder meetings preceded Quinn and Kumar's 2014 publication, many of them can be classified according to this framework.^8^ Proximal causes that influence differential exposure are addressed in Bouye et al.^28^ and Hutchins et al.,^29^ whereas proposals to target distal determinants are proposed in Truman et al.,^31^ and Steege et al.^30^

While many intervention studies included in this review focus on natural disaster preparedness and response, they represent real-world applications of the influenza pandemic recommendations outlined in the 2008 CDC stakeholder meetings. For instance, the intervention studied in Eisenman et al.^13^ demonstrates the positive effect that interpersonal, culturally appropriate education delivered by a community health worker can have on disaster preparedness in marginalized communities. These results validate the recommendation in Steege et al.^30^ for using lay *promotoras* in delivery of health services, goods, and messaging in the case of a pandemic. These results could be translated into an intervention to reach marginalized populations during the current COVID-19 pandemic.

The importance of community engagement and partnership with community leaders was repeated often in expert-based recommendations and was empirically grounded in some studies we examined.^29^ In Aten et al., the authors found that AA/Black clergy could be integral as community liaisons during Hurricane Katrina They also found that churches could serve as sites for delivery of community-based services.^19,20^ Such partnerships are crucial to lessen the disproportionate burden of COVID-19 in racial and ethnic minorities. While it is too early in the pandemic to expect a rigorous evaluation of the effect of partnerships with faith-based organizations on COVID-19 disparities, the popular media have already chronicled that such interventions are underway.^35^

The interventions described in this study emphasize that preparedness efforts must be prioritized, and marginalized communities must be included *before* disaster hits. Nevertheless, some of the lessons learned may be relevant to the current pandemic phase. Proven preparedness interventions could also be considered for implementation now given the potential for future waves of COVID-19 or new epidemics to emerge.

What remains missing from the studies in this review are examples of successful system-level interventions that target the distal determinants of worse outcomes in a pandemic in marginalized populations. This gap persists despite evidence from the H1N1 pandemic that variables of exposure that occur at higher rates among these disadvantaged groups, such as inability to take sick leave, can drastically affect disease rates.^36^

In the key stakeholder reports, we find multiple system-level recommendations, for instance liberal workplace leave and teleworking policies,^31^ wage freezes and childcare vouchers,^28^ and creating an ethical and equitable system for ensuring access to treatment and vaccination, particularly among the uninsured.^29^ Some of these interventions are underway, and researchers should actively test their impact on health disparities so that lessons learned may be applied to our current and possible future pandemics.

This review has some limitations. Given the limited number of studies on infectious disease interventions that fit criteria for inclusion, we decided to also include studies on disaster response interventions, which may not be as generalizable to the current context of COVID-19. Additionally, most of the studies reviewed were observational or qualitative in nature, and therefore did not include the rigor that randomization and/or a longitudinal design may lend.

## Conclusion

Relatively few studies have examined interventions to mitigate health disparities during a disease pandemic. The studies and expert recommendations included in this review focus largely on the importance of preparedness before onset of a disaster or pandemic to prevent disproportionate impact on marginalized populations. A few studies included interventions that could be adapted to the current COVID-19 pandemic, including offering childcare support, partnering with trusted community liaisons to deliver important messaging around disease mitigation, and using culturally specific communication to best reach marginalized groups. To better prevent widespread health disparities that emerge in the wake of the current and future infectious disease epidemics, more research is needed on policy and system-level interventions and their effect on the distal determinants of poor health outcomes among marginalized groups.

## Supplementary Material

Supplemental data

## Supplementary Material

Supplementary Appendix SA1

Supplementary Appendix SA2

Supplementary Appendix SA3

Supplementary Appendix SA4

Supplementary Appendix Table ST1

## Abbreviations Used

AA: African American
AED: Automated external defibrillator
AI/AN: American Indian/Alaska Native
C: control
CASP: Critical Appraisal Skills Program
CBO: community-based organization
CDC: Centers for Disease Control
CI: confidence interval
CPR: cardiopulmonary resuscitation
EMR: electronic medical record
EMS: emergency medical services
FBO: faith-based organization
HBPC: Home-based Primary Care
I: intervention
LA: Los Angeles
LEP: limited English proficiency
MSFW: migrant and seasonal farmworkers
N/A: not applicable
NCID: National Center for Infectious Disease
OR: odds ratio
OT: occupational therapy
PH: public health
PI: Pacific Islander
PPE: personal protective equipment
RCT: randomized controlled trial
SARS: severe acute respiratory syndrome
SD: standard deviation
SES: socioeconomic status
US: United States
VHA: Veterans Health Administration
