# Supplementary material for: Preventing Unequal Health Outcomes in COVID-19: A Systematic Review of Past Interventions
Source: Health Equity. 2021 Dec 27;5(1):856–71. doi: 10.1089/heq.2021.0016 (PMC8742307; doi:10.1089/heq.2021.0016)
Supplement: Supplemental data [file Supp_App.docx]

**Preventing Unequal Health Outcomes in COVID-19: A Systematic Review of Past Interventions**

**APPENDICES**

[Appendix Item 1. Search Strategies (Parent VA Review) 2](#_Toc60845222)

[Appendix Item 2. Study Selection Criteria (Parent VA Review) 9](#_Toc60845223)

[Appendix Item 3. Quality Ratings 12](#_Toc60845224)

[Table. Quality Ratings for Cross-sectional Studies 12](#_Toc60845225)

[Table. Quality Ratings for Cohort Studies 13](#_Toc60845226)

[Table. Quality Ratings for Qualitative Studies 14](#_Toc60845227)

[Appendix Table 1. PICOTS 15](#_Toc60845228)

[Appendix References 17](#_Toc60845229)

Appendix Item 1. Search Strategies (Parent VA Review)

**Ovid MEDLINE ALL 1946 to May 01, 2020**

Date searched: May 4, 2020

1 Coronavirus Infections/ or COVID-19.rs. (6243)

2 ((("Corona virinae" or "corona virus" or Coronavirinae or coronavirus or COVID or nCoV) adj4 ("19" or "2019" or novel or new)) or (("Corona virinae" or "corona virus" or Coronavirinae or coronavirus or COVID or nCoV) and (wuhan or china or chinese)) or "Corona virinae19" or "Corona virinae2019" or "corona virus19" or "corona virus2019" or Coronavirinae19 or Coronavirinae-19 or Coronavirinae2019 or Coronavirinae-2019 or coronavirus19 or coronavirus-19 or coronavirus2019 or coronavirus-2019 or COVID19 or COVID-19 or COVID2019 or COVID-2019 or nCOV19 or nCOV-19 or nCOV2019 or nCOV-2019 or SARS-CoV-2 or SARS-CoV2 or SARS-CoV or "severe acute respiratory").ti,ab,hw,kw. (16846)

3 or/1-2 (20340)

4 Healthcare Disparities/ or Health Equity/ or Health Status Disparities/ or Culturally Competent Care/ or Social Determinants of Health/ or Sociology, Medical/ (35656)

5 (disadvantaged or discriminat* or disparat* or disparit* or disproportion* or inequal* or inequit* or unequal or underserved or under-served or (cultural* adj3 compet*) or (social* adj3 determin*)).ti,ab,kf. or (difference or different).ti. (679237)

6 Ethnic Groups/ or Minority Groups/ or African Americans/ or Arabs/ or Asian Americans/ or Hispanic Americans/ or Mexican Americans/ or Indigenous Peoples/ or exp Alaska Natives/ (152741)

7 (ethnic* or race* or racial* or minority or minorities or "people of color" or African-American* or Black or Blacks or Hispanic* or Chican* or Latino* or Latina* or Latinx or Mexican-American* or Asian-American* or Chinese-American or Filipino* or Japanese or Korean or Vietnamese or Native American* or Indian or Indians or indigenous).ti,ab,kf. (711222)

8 Socioeconomic Factors/ or Economic Status/ or exp Employment/ or Homeless Persons/ or Medicaid/ or Medically Uninsured/ or exp Medicare/ or Poverty/ or Poverty Areas/ or Public Assistance/ or Public Housing/ or Social Class/ or "Transients and Migrants"/ or Undocumented Immigrants/ or Veterans/ or Working Poor/ (388391)

9 ("blue collar" or impoverish* or homeless* or immigrant* or indigent or low-income or low-wage or lower-income or Medicaid or Medicare or migrant* or poverty or (public adj (assistance or housing)) or social or socio* or SES or undocumented or uninsured or veteran* or (working adj2 (class or poor))).ti,ab,kf. (876545)

10 Disabled Persons/ or Health Services for Persons with Disabilities/ or Persons With Hearing Impairments/ or Visually Impaired Persons/ or Vulnerable Populations/ (56412)

11 (disabilit* or disabled or blind or deaf or handicapped or ((visual* or hearing or physical*) adj impair*) or vulnerab*).ti,ab,kf. (557881)

12 Rural Health/ or Rural Health Services/ or Rural Population/ or Urban Health/ or Urban Health Services/ or Urban Population/ 146477)

13 (inner-city or metropol* or municipal* or neighborhood* or rural-urban or rural or urban or "New York" or "Los Angeles" or Chicago or Houston or Phoenix of Philadelphia or "San Antonio" or "San Diego" or Dallas or "San Jose" or "San Francisco" or Austin or Jacksonville or "Fort Worth" or Columbus or Charlotte or Indianapolis or Seattle or Denver or Washington or Boston or "El Paso" or Detroit or Nashville or Portland or Memphis or "Oklahoma City" or "Las Vegas" or Louisville or Baltimore or Milwaukee or Albuquerque or Tucson or Fresno or Mesa or Sacramento or Atlanta or "Kansas City" or "Colorado Springs" or Miami or Raleigh or Omaha or "Long Beach" or "Virginia Beach" or Oakland or Minneapolis or Tulsa or Arlington or Tampa or "New Orleans").ti,ab,kf. or (city* or cities or county).ti,kf. (530985)

14 or/4-13 (3076764)

15 3 and 14 (1963)

16 15 not (Beijing or "Hong Kong" or Huangshi or Hubei or Jiangsu or London or Paris or Qingdao or Shanghai or Shenzhen or Tianjin or "Wuhan city" or Zhuhai or Caribbean or Europe* or "South America" or "South Asia" or "Southeast Asia" or "East Asia" or "North Africa" or "East Africa" or "West Africa" or "Southern Africa" or Afghanistan or Albania* or Algeria* or Andorra or Angola or Antigua or Argentin* or Armenia* or Australia* or Austria* or Azerbaijan or Bahamas or Bahrain or Bangladesh* or Barbados or Belarus or Belgium or Belize or Benin or Bhutan* or Bolivia* or Bosnia* or Botswana or Brazil* or Britain* or Brunei or Bulgaria* or "Burkina Faso" or Burundi or "Cabo Verde" or Cambodia* or Cameroon* or Canada or Canadian* or "Central African Republic" or Chad or Chile* or China or Colombia* or Comoros or Congo* or "Costa Rica" or "Cote d'Ivoire" or Croatia* or Cuba or Cyprus or Czechia or Denmark or Djibouti or Dominica or "Dominican Republic" or Ecuador or Egypt* or "El Salvador" or England or "Equatorial Guinea" or Eritrea* or Estonia* or Eswatini or Ethiopia* or Fiji* or Finland or France or French or Gabon or Gambia* or Germany or German* or Ghana or Greece or Grenada or Guatemala* or Guinea or Guinea-Bissau or Guyana or Haiti* or Honduras or Hungary or Iceland or India or Indonesia* or Iran* or Iraq* or Ireland or Israel* or Italy or Jamaica* or Japan or Jordan* or Kazakhstan or Kenya* or Kiribati or Korea or Kosovo or Kuwait or Kyrgyzstan or Laos or Latvia* or Lebanon or Lesotho or Liberia* or Libya* or Liechtenstein or Lithuania* or Luxembourg or Madagascar or Malawi or Malaysia* or Maldives or Mali or Malta or "Marshall Islands" or Mauritania* or Mauritius or Mexico or Micronesia* or Moldova or Monaco or Mongolia* or Montenegro or Morocco or Mozambique or Myanmar or Namibia* or Nauru or Nepal* or Netherlands or "New Zealand" or Nicaragua* or Niger or Nigeria* or Macedonia* or Norway or Oman or Pakistan or Palau or Palestine or Panama or "Papua New Guinea" or Paraguay or Peru or Philippines or Poland or Portugal or Qatar or Romania* or Russia* or Rwanda* or "Saint Kitts" or "Saint Lucia" or "Saint Vincent" or "San Marino" or "Sao Tome" or Saudi* or Scotland or Senegal* or Serbia* or Seychelles or "Sierra Leone" or Singapore* or Slovakia* or Slovenia* or "Solomon Islands" or Somalia* or "South Africa" or "South Sudan" or Spain or "Sri Lanka" or Sudan* or Suriname or Sweden or Switzerland or Syria* or Taiwan* or Tajikistan or Tanzania* or Thailand or "Timor-Leste" or "East Timor" or Togo or Tonga* or Trinidad or Tunisia* or Turkey or Turkmenistan or Tuvalu or Uganda* or Ukraine or "United Arab Emirates" or "United Kingdom" or UK or Uruguay or Uzbekistan or Vanuatu or Venezuela* or Vietnam or Yemen or Zambia* or Zimbabwe*).ti. (1391)

17 limit 16 to english language (1295)

18 limit 17 to yr="2020 -Current" (637)

**Ovid PsycINFO 1806 to May Week 1 2020**

Date searched: May 8, 2020

1 *Pandemics/ or *Epidemics/ or *Disasters/ or *Natural Disasters/ or Emergency Preparedness/ (10503)

2 (disaster* or earthquake* or epidemic* or hurricane* or pandemic* or postdisaster or post-disaster or (public adj3 emergenc*) or H1N1 or SARS or Zika or "severe acute respiratory").ti. (9540)

3 or/1-2 (13615)

4 *Health Disparities/ (6152)

5 (difference* or disadvantaged or discriminat* or disparat* or disparit* or disproportion* or inequal* or inequit* or unequal or underserved or under-served or (cultural* adj3 compet*) or (social* adj3 determin*)).ti. (116879)

6 *"Racial and Ethnic Differences"/ or *"Race and Ethnic Discrimination"/ or *Ethnic Groups/ or *Minority Groups/ or *Alaska Natives/ or *American Indians/ or *Arabs/ or exp *Asians/ or *Blacks/ or *Hawaii Natives/ or *Jews/ or exp *"Latinos/Latinos"/ or *Pacific Islanders/ (92858)

7 (ethnic* or race* or racial* or minority or minorities or "people of color" or African-American* or Black or Blacks or Hispanic* or Chican* or Latino* or Latina* or Latinx or Mexican-American* or Asian-American* or Chinese-American or Filipino* or Japanese or Korean or Vietnamese or Native American* or Indian or Indians or indigenous).ti. (130827)

8 *Disadvantaged/ or *Socioeconomic Status/ or *Lower Class/ or *Lower Income Level/ or *Poverty/ or *Social Class/ or exp *Homeless/ or *Shelters/ or *Social Services/ or *"Uninsured (Health Insurance)"/ or *"Welfare Services (Government)"/ or *Immigration/ or *Migrant Farm Workers/ or *Refugees/ (78171)

9 ("blue collar" or impoverish* or homeless* or immigrant* or indigent or low-income or low-wage or lower-income or Medicaid or Medicare or migrant* or immigrant* or poverty or "public assistance" or "public housing" or socio* or SES or undocumented or uninsured or veteran* or "working class" or "working poor").ti. (75944)

10 exp *Disabilities/ or *Disability Discrimination/ or *"At Risk Populations"/ or *Blind/ or *Deaf/ or *Partially Hearing Impaired/ (84942)

11 (disabilit* or disabled or blind or deaf or handicapped or ((visual* or hearing or physical*) adj impair*) or vulnerab*).ti. (88840)

12 exp *Neighborhoods/ or *Rural Environments/ or *Urban Environments/ (28918)

13 (city or cities or county or inner-city or metropol* or municipal* or neighborhood* or rural-urban or rural or urban or "New York" or "Los Angeles" or Chicago or Houston or Phoenix of Philadelphia or "San Antonio" or "San Diego" or Dallas or "San Jose" or "San Francisco" or Austin or Jacksonville or "Fort Worth" or Columbus or (Charlotte and (SC or Carolina*)) or Indianapolis or Seattle or Denver or Washington or Boston or "El Paso" or Detroit or Nashville or Portland or Memphis or "Oklahoma City" or "Las Vegas" or Louisville or Baltimore or Milwaukee or Albuquerque or Tucson or Fresno or Mesa or Sacramento or Atlanta or "Kansas City" or "Colorado Springs" or Miami or Raleigh or Omaha or "Long Beach" or "Virginia Beach" or Oakland or Minneapolis or Tulsa or Arlington or Tampa or "New Orleans").ti. (59491)

14 or/4-13 (543361)

15 3 and 14 (1823)

16 15 not (Beijing or "Hong Kong" or Huangshi or Hubei or Jiangsu or London or Paris or Qingdao or Shanghai or Shenzhen or Tianjin or "Wuhan city" or Zhuhai or Kathmandu).ti. (1816)

17 limit 16 to english language (1751)

18 17 not (epidemic* adj2 (cigarette or cocaine or crack or diabetes or heroin or obesity or opioid or opioids or smoking or tobacco)).ti,ab.(1701)

19 18 not (cat or cats or dog or dogs or mice or mouse or rat or rats or rodent).ti.(1700)

20 19 not (Caribbean or Europe or "South America" or "South Asia" or "Southeast Asia" or "East Asia" or "North Africa" or "East Africa" or "West Africa" or "Southern Africa" or Afghanistan or Albania or Algeria or Andorra or Angola or Antigua or Argentina Armenia or Australia or Austria or Azerbaijan or Bahamas or Bahrain or Bangladesh or Barbados or Belarus or Belgium or Belize or Benin or Bhutan or Bolivia or Bosnia or Botswana or Brazil or Brunei or Bulgaria or "Burkina Faso" or Burundi or "Cabo Verde" or Cambodia or Cameroon or Canada or "Central African Republic" or Chad or Chile or China or Colombia or Comoros or Congo or "Costa Rica" or "Cote d'Ivoire" or Croatia or Cuba or Cyprus or Czechia or Denmark or Djibouti or Dominica or "Dominican Republic" or Ecuador or Egypt or "El Salvador" or "Equatorial Guinea" or Eritrea or Estonia or Eswatini or Ethiopia or Fiji or Finland or France or Gabon or Gambia or Georgia or Germany or Ghana or Greece or Grenada or Guatemala or Guinea or Guinea-Bissau or Guyana or Haiti or Honduras or Hungary or Iceland or India or Indonesia or Iran or Iraq or Ireland or Israel or Italy or Jamaica or Japan or Jordan or Kazakhstan or Kenya or Kiribati or Korea or Kosovo or Kuwait or Kyrgyzstan or Laos or Latvia or Lebanon or Lesotho or Liberia or Libya or Liechtenstein or Lithuania or Luxembourg or Madagascar or Malawi or Malaysia or Maldives or Mali or Malta or "Marshall Islands" or Mauritania or Mauritius or Mexico or Micronesia or Moldova or Monaco or Mongolia or Montenegro or Morocco or Mozambique or Myanmar or Namibia or Nauru or Nepal or Netherlands or "New Zealand" or Nicaragua or Niger or Nigeria or Macedonia or Norway or Oman or Pakistan or Palau or Palestine or Panama or "Papua New Guinea" or Paraguay or Peru or Philippines or Poland or Portugal or Qatar or Romania or Russia or Rwanda or "Saint Kitts" or "Saint Lucia" or "Saint Vincent" or "San Marino" or "Sao Tome" or Saudi or Senegal or Serbia or Seychelles or Sierra Leone or Singapore or Slovakia or Slovenia or "Solomon Islands" or Somalia or "South Africa" or "South Sudan" or Spain or "Sri Lanka" or Sudan or Suriname or Sweden or Switzerland or Syria or Taiwan or Tajikistan or Tanzania or Thailand or "Timor-Leste" or "East Timore" or Togo or Tonga or Trinidad or Tunisia or Turkey or Turkmenistan or Tuvalu or Uganda or Ukraine or "United Arab Emirates" or "United Kingdom" or UK or Uruguay or Uzbekistan or Vanuatu or Venezuela or Vietnam or Yemen or Zambia or Zimbabwe).ti,lo. (1165)

**EBM Reviews - Cochrane Central Register of Controlled Trials April 2020**

Date searched: May 11, 2020

1 (disaster* or earthquake* or epidemic* or hurricane* or pandemic* or post-disaster or (public adj3 emergenc*) or H1N1 or SARS or Zika or "severe acute respiratory").ti. (1455)

2 (difference* or disadvantaged or discriminat* or disparat* or disparit* or disproportion* or inequal* or inequit* or unequal or underserved or under-served or (cultural* adj3 compet*) or (social* adj3 determin*)).ti. (8858)

3 (ethnic* or race* or racial* or minority or minorities or "people of color" or African-American* or Black or Blacks or Hispanic* or Chican* or Latino* or Latina* or Latinx or Mexican-American* or Asian-American* or Chinese-American or Filipino* or Japanese or Korean or Vietnamese or Native American* or Indian or Indians or indigenous).ti. (18233)

4 ("blue collar" or impoverish* or homeless* or immigrant* or indigent or low-income or low-wage or lower-income or Medicaid or Medicare or migrant* or poverty or (public adj (assistance or housing)) or social or socio* or SES or undocumented or uninsured or veteran* or (working adj2 (class or poor))).ti. (13475)

5 (disabilit* or disabled or blind or deaf or handicapped or ((visual* or hearing or physical*) adj impair*) or vulnerab*).ti. (87945)

6 (city or cities or county or inner-city or metropol* or municipal* or neighborhood* or rural-urban or rural or urban or "New York" or "Los Angeles" or Chicago or Houston or Phoenix of Philadelphia or "San Antonio" or "San Diego" or Dallas or "San Jose" or "San Francisco" or Austin or Jacksonville or "Fort Worth" or Columbus or (Charlotte and (SC or Carolina*)) or Indianapolis or Seattle or Denver or Washington or Boston or "El Paso" or Detroit or Nashville or Portland or Memphis or "Oklahoma City" or "Las Vegas" or Louisville or Baltimore or Milwaukee or Albuquerque or Tucson or Fresno or Mesa or Sacramento or Atlanta or "Kansas City" or "Colorado Springs" or Miami or Raleigh or Omaha or "Long Beach" or "Virginia Beach" or Oakland or Minneapolis or Tulsa or Arlington or Tampa or "New Orleans").ti. (8380)

7 or/2-6 (132248)

8 and/1,7 (112)

9 8 not (Beijing or "Hong Kong" or Huangshi or Hubei or Jiangsu or London or Paris or Qingdao or Shanghai or Shenzhen or Tianjin or "Wuhan city" or Zhuhai or Australia* or Bangladesh or Britain or Canada or China or Europe* or England or France or India or Iran or Ireland or Italy or Japan or Korea or Pakistan or Singapore or Scotland or "South Korea" or Thailand or Turkey or "United Kingdom" or UK).ti. (104)

10 9 not (epidemic* adj2 (cigarette or cocaine or crack or diabetes or heroin or obesity or opioid or opioids or smoking or tobacco)).ti,ab. (104)

11 10 not (cat or cats or dog or dogs or mice or mouse or rat or rats or rodent).ti. (104)

12 11 not (Caribbean or Europe or "South America" or "South Asia" or "Southeast Asia" or "East Asia" or "North Africa" or "East Africa" or "West Africa" or "Southern Africa" or Afghanistan or Albania or Algeria or Andorra or Angola or Antigua or Argentina Armenia or Australia or Austria or Azerbaijan or Bahamas or Bahrain or Bangladesh or Barbados or Belarus or Belgium or Belize or Benin or Bhutan or Bolivia or Bosnia or Botswana or Brazil or Brunei or Bulgaria or "Burkina Faso" or Burundi or "Cabo Verde" or Cambodia or Cameroon or Canada or "Central African Republic" or Chad or Chile or China or Colombia or Comoros or Congo or "Costa Rica" or "Cote d'Ivoire" or Croatia or Cuba or Cyprus or Czechia or Denmark or Djibouti or Dominica or "Dominican Republic" or Ecuador or Egypt or "El Salvador" or "Equatorial Guinea" or Eritrea or Estonia or Eswatini or Ethiopia or Fiji or Finland or France or Gabon or Gambia or Georgia or Germany or Ghana or Greece or Grenada or Guatemala or Guinea or Guinea-Bissau or Guyana or Haiti or Honduras or Hungary or Iceland or India or Indonesia or Iran or Iraq or Ireland or Israel or Italy or Jamaica or Japan or Jordan or Kazakhstan or Kenya or Kiribati or Korea or Kosovo or Kuwait or Kyrgyzstan or Laos or Latvia or Lebanon or Lesotho or Liberia or Libya or Liechtenstein or Lithuania or Luxembourg or Madagascar or Malawi or Malaysia or Maldives or Mali or Malta or "Marshall Islands" or Mauritania or Mauritius or Mexico or Micronesia or Moldova or Monaco or Mongolia or Montenegro or Morocco or Mozambique or Myanmar or Namibia or Nauru or Nepal or Netherlands or "New Zealand" or Nicaragua or Niger or Nigeria or Macedonia or Norway or Oman or Pakistan or Palau or Palestine or Panama or "Papua New Guinea" or Paraguay or Peru or Philippines or Poland or Portugal or Qatar or Romania or Russia or Rwanda or "Saint Kitts" or "Saint Lucia" or "Saint Vincent" or "San Marino" or "Sao Tome" or Saudi or Senegal or Serbia or Seychelles or Sierra Leone or Singapore or Slovakia or Slovenia or "Solomon Islands" or Somalia or "South Africa" or "South Sudan" or Spain or "Sri Lanka" or Sudan or Suriname or Sweden or Switzerland or Syria or Taiwan or Tajikistan or Tanzania or Thailand or "Timor-Leste" or "East Timore" or Togo or Tonga or Trinidad or Tunisia or Turkey or Turkmenistan or Tuvalu or Uganda or Ukraine or "United Arab Emirates" or "United Kingdom" or UK or Uruguay or Uzbekistan or Vanuatu or Venezuela or Vietnam or Yemen or Zambia or Zimbabwe).ti. (95)

**EBM Reviews - Cochrane Central Register of Controlled Trials April 2020**

Date searched: May 11, 2020

1 (disaster* or earthquake* or epidemic* or hurricane* or pandemic* or post-disaster or (public adj3 emergenc*) or H1N1 or SARS or Zika or "severe acute respiratory").ti. (1455)

2 (difference* or disadvantaged or discriminat* or disparat* or disparit* or disproportion* or inequal* or inequit* or unequal or underserved or under-served or (cultural* adj3 compet*) or (social* adj3 determin*)).ti. (8858)

3 (ethnic* or race* or racial* or minority or minorities or "people of color" or African-American* or Black or Blacks or Hispanic* or Chican* or Latino* or Latina* or Latinx or Mexican-American* or Asian-American* or Chinese-American or Filipino* or Japanese or Korean or Vietnamese or Native American* or Indian or Indians or indigenous).ti. (18233)

4 ("blue collar" or impoverish* or homeless* or immigrant* or indigent or low-income or low-wage or lower-income or Medicaid or Medicare or migrant* or poverty or (public adj (assistance or housing)) or social or socio* or SES or undocumented or uninsured or veteran* or (working adj2 (class or poor))).ti. (13475)

5 (disabilit* or disabled or blind or deaf or handicapped or ((visual* or hearing or physical*) adj impair*) or PTSD or post-traumatic stress or posttraumatic stress or vulnerab* or ((severe* or serious* or chronic* or persistent*) adj mental* ill*)).ti. (91871)

6 (city or cities or county or inner-city or metropol* or municipal* or neighborhood* or rural-urban or rural or urban or "New York" or "Los Angeles" or Chicago or Houston or Phoenix of Philadelphia or "San Antonio" or "San Diego" or Dallas or "San Jose" or "San Francisco" or Austin or Jacksonville or "Fort Worth" or Columbus or (Charlotte and (SC or Carolina*)) or Indianapolis or Seattle or Denver or Washington or Boston or "El Paso" or Detroit or Nashville or Portland or Memphis or "Oklahoma City" or "Las Vegas" or Louisville or Baltimore or Milwaukee or Albuquerque or Tucson or Fresno or Mesa or Sacramento or Atlanta or "Kansas City" or "Colorado Springs" or Miami or Raleigh or Omaha or "Long Beach" or "Virginia Beach" or Oakland or Minneapolis or Tulsa or Arlington or Tampa or "New Orleans").ti. (8380)

7 or/2-6 (135518)

8 and/1,7 (138)

9 8 not (Beijing or "Hong Kong" or Huangshi or Hubei or Jiangsu or London or Paris or Qingdao or Shanghai or Shenzhen or Tianjin or "Wuhan city" or Zhuhai or Australia* or Bangladesh or Britain or Canada or China or Europe* or England or France or India or Iran or Ireland or Italy or Japan or Korea or Pakistan or Singapore or Scotland or "South Korea" or Thailand or Turkey or "United Kingdom" or UK).ti. (126)

10 9 not (epidemic* adj2 (cigarette or cocaine or crack or diabetes or heroin or obesity or opioid or opioids or smoking or tobacco)).ti,ab. (126)

11 10 not (cat or cats or dog or dogs or mice or mouse or rat or rats or rodent).ti. (126)

12 11 not (Caribbean or Europe or "South America" or "South Asia" or "Southeast Asia" or "East Asia" or "North Africa" or "East Africa" or "West Africa" or "Southern Africa" or Afghanistan or Albania or Algeria or Andorra or Angola or Antigua or Argentina Armenia or Australia or Austria or Azerbaijan or Bahamas or Bahrain or Bangladesh or Barbados or Belarus or Belgium or Belize or Benin or Bhutan or Bolivia or Bosnia or Botswana or Brazil or Brunei or Bulgaria or "Burkina Faso" or Burundi or "Cabo Verde" or Cambodia or Cameroon or Canada or "Central African Republic" or Chad or Chile or China or Colombia or Comoros or Congo or "Costa Rica" or "Cote d'Ivoire" or Croatia or Cuba or Cyprus or Czechia or Denmark or Djibouti or Dominica or "Dominican Republic" or Ecuador or Egypt or "El Salvador" or "Equatorial Guinea" or Eritrea or Estonia or Eswatini or Ethiopia or Fiji or Finland or France or Gabon or Gambia or Georgia or Germany or Ghana or Greece or Grenada or Guatemala or Guinea or Guinea-Bissau or Guyana or Haiti or Honduras or Hungary or Iceland or India or Indonesia or Iran or Iraq or Ireland or Israel or Italy or Jamaica or Japan or Jordan or Kazakhstan or Kenya or Kiribati or Korea or Kosovo or Kuwait or Kyrgyzstan or Laos or Latvia or Lebanon or Lesotho or Liberia or Libya or Liechtenstein or Lithuania or Luxembourg or Madagascar or Malawi or Malaysia or Maldives or Mali or Malta or "Marshall Islands" or Mauritania or Mauritius or Mexico or Micronesia or Moldova or Monaco or Mongolia or Montenegro or Morocco or Mozambique or Myanmar or Namibia or Nauru or Nepal or Netherlands or "New Zealand" or Nicaragua or Niger or Nigeria or Macedonia or Norway or Oman or Pakistan or Palau or Palestine or Panama or "Papua New Guinea" or Paraguay or Peru or Philippines or Poland or Portugal or Qatar or Romania or Russia or Rwanda or "Saint Kitts" or "Saint Lucia" or "Saint Vincent" or "San Marino" or "Sao Tome" or Saudi or Senegal or Serbia or Seychelles or Sierra Leone or Singapore or Slovakia or Slovenia or "Solomon Islands" or Somalia or "South Africa" or "South Sudan" or Spain or "Sri Lanka" or Sudan or Suriname or Sweden or Switzerland or Syria or Taiwan or Tajikistan or Tanzania or Thailand or "Timor-Leste" or "East Timore" or Togo or Tonga or Trinidad or Tunisia or Turkey or Turkmenistan or Tuvalu or Uganda or Ukraine or "United Arab Emirates" or "United Kingdom" or UK or Uruguay or Uzbekistan or Vanuatu or Venezuela or Vietnam or Yemen or Zambia or Zimbabwe).ti. (117)

Appendix Item 2. Study Selection Criteria (Parent VA Review)

1. **Language**: Is the full text of the article in English?

Yes ⭢ Proceed to #2

No ⭢ Code **X1** (NA Language). STOP

1. **Population**: Are participants Adults?

*In mixed-age studies, findings must be reported separately for adults.*

Yes ⭢ Proceed to #3

No ⭢ **Code** **X2** (NA population). Add code **B** if retaining for background/discussion. STOP

1. **Population**: Does the study provide data specific to or stratified by 1 or more of the following participant populations: racial/ethnic minorities or persons a) with disabilities; b) of low socioeconomic status; c) living in rural communities; d) living in population dense neighborhoods; e) living in high-poverty neighborhoods.

*Note: For SES, proxies may include occupation, education, neighborhoods or geographic regions, income, insurance-status, Medicare/Medicaid, SNAP, TANF, households with children qualifying for free or reduced school lunch,*

Yes ⭢ Proceed to #4

No ⭢ **Code X3** (NA subpopulation)**.** Add code **B** if retaining for background/discussion. STOP

1. **Setting**: Is this a US-only population (including U.S territories)?

Yes ⭢ Proceed to #5

No ⭢ **Code X4** (NA setting). Add code **B** if retaining for background/discussion. STOP

1. **Timing**: Is the study performed during or in preparation or response to an infectious disease pandemic or epidemic, or a disaster?

Yes ⭢ Proceed to #6

No ⭢ Code **X5** (NA timing)**.** Add code **B** if retaining for background/discussion. STOP

1. **Study design and type**: Is the article an original research study, or a systematic review/meta-analysis?

*Excluded: Dissertations, non-systematic reviews, conference abstracts, protocols, erratum, comments, non-research letters*

Yes ⭢ Proceed to #7

No ⭢ Code **X6** (NA study design/type)**.** Add code **B** if retaining for background/discussion. STOP

1. **Outcomes**: Is the outcome health-related?

*Examples: Utilization or access, infection, burden of illness, severity, mortality, morbidity*

*Note: Outcomes may also be factors that contribute to disparities in health-related outcome (eg, trust)*

Yes ⭢ Proceed to #9

No ⭢ Proceed to #8

1. **Outcomes**: Is the outcome a result of measures implemented during a pandemic, epidemic, or disaster (*eg,* social distancing, school closure, etc.) that impact a health-related outcome?

*Examples: Employment loss or reduction, income reduction*

Yes ⭢ Proceed to #9

No ⭢ **Code X7** (NA outcome). Add code **B** if retaining for background/discussion. STOP

1. **Comparator**: Does the study include a comparison (within the same population or to a relevant comparator); or, if an intervention study, does it compare results to no intervention, pre-intervention, or another intervention or public health response?

Yes ⭢ Proceed to #11

No ⭢ Proceed to #10

1. **Pre-intervention studies**: Does the study inform a future intervention (*eg,* survey, qualitative study)

*Examples:* *Preferred forms of communication, emergency preparedness, social distancing, access to care*

Yes ⭢ Proceed to #11

No ⭢ **Code X8** (NA comparator). Add code **B** if retaining for background/discussion. STOP

1. **KQ1**: Does the study examine the COVID-19 Pandemic?

Yes ⭢ **Code KQ1.** If the study is a systematic review or meta-analysis, **Code KQ1SR**.

STOP

No ⭢ Proceed to #12

1. **Timing**: Is the study performed during, or in preparation or response to, a disaster?

Yes ⭢ Proceed to #14

No ⭢ Proceed to #13

1. **KQ2** **Contributing Factors:** Does the study examine factors that **contribute to** health inequalities?

*Examples: Risk of exposure, susceptibility/risk of poor outcomes, access to care, trust in healthcare system, discrimination.*

*Note: These are not 1^st^ Generation/Phase I studies that simply find that a disparity exists. These are 2^nd^ Generation/Phase II studies that help us to understand why (see Kilbourne et. al, 2006 – uploaded to Slack).*

Yes ⭢ **Code KQ2.** If the study is a systematic review or meta-analysis, **Code KQ2SR**. Proceed to #14

No ⭢ proceed to #14

1. **KQ3**: Is the study a 1) program evaluation, pre-intervention study (*eg,* survey, qualitative), or an intervention study 2) designed to mitigate health inequalities?

*Select “Yes” only if both 1) and 2) are true*

Yes ⭢ If the study is not also included in KQ2, **Code KQ3.** If the study is a systematic review or meta-analysis, **Code KQ3SR**.

Yes ⭢ If the study is also included in KQ2, **Code KQ2&3.** If the study is a systematic review or meta-analysis, **Code KQ2&3SR**.

No ⭢ **Code X9** (NA factors or intervention)**.** Add code **B** if retaining for background/discussion. STOP

*Note: B codes can be added for any excluded study that we should retain/reference for background or discussion.*

**Key Questions**

KQ1: In the COVID-19 pandemic:

1. What health inequalities have been described?
2. What factors have contributed to health inequalities?
3. What is the effectiveness of interventions used to address health inequalities?

KQ2: What factors contribute to disparate infection rates and health-related outcomes among different segments of the population during infectious disease epidemics or pandemics?

KQ3: What interventions have been used to reduce health inequalities in infectious disease transmission or health outcomes in disasters, epidemics or pandemics?

**Codes Key:**

X1: NA Language

X2: NA Population

X3: NA Subpopulation

X4: NA Setting

X5: NA Timing

X6: NA Study design/type

X7: NA Outcome

X8: NA Comparator

X9: NA factor/intervention

Appendix Item 3. Quality Ratings

Table. Quality Ratings for Cross-sectional Studies

| **Author** | **1** | **2** | **3** | **4** | **5** | **6** | **7** | **Quality concerns** | **Applicability(1)** |
| --- | --- | --- | --- | --- | --- | --- | --- | --- | --- |
| Price, 2013(2) | Y | Y | Y | Y | Y, A&B | Y | Y | -- | Fair |
| Wyte-Lake, 2019(3) | U | Y | NA | U | N | P | N | No control for confounders, methods poorly reported | Poor |

Abbreviations: N=No; NA=Not applicable; NR=Not reported; P=Partial; U=Unclear; Y=Yes

Criteria (Adapted Newcastle-Ottawa(4)):

Selection

1. Sample representative?

yes = Truly representative of the average in the target population (all subjects or random sampling); or, somewhat representative of the average in the target population. (non-random sampling)

no = Selected group of users

unclear = No description of the sampling strategy.

1. Sample size justified and satisfactory?

Yes/no

1. Non-respondents comparable to respondents?

yes = Comparability between respondents and non-respondents characteristics is established, and the response rate is satisfactory.

no = The response rate is unsatisfactory, or the comparability between respondents and non-respondents is unsatisfactory.

unclear = No description of the response rate or the characteristics of the responders and the non-responders.

1. Ascertainment of the exposure (risk factor) appropriate?

yes = Adequately described

unclear = Not adequate description of the measurement tool.

Comparability

1. The subjects in different outcome groups are comparable, based on the study design or analysis. Confounding factors are controlled.

yes = specify a, b, or a&b

a) The study controls for the most important factor (age).

b) The study control for any additional factor.

no = no adjustment for potential confounders

Outcome

1. Assessment of the outcome appropriate?

Yes = Independent blind assessment or Record linkage.

Partial = Self report.

unclear = No description.

1. Statistical test described and appropriate (including measurement of the association, including CIs and probability level [p- value])?

Yes/no/unclear

Table. Quality Ratings for Cohort Studies

| **Study** | **1** | **2** | **3** | **4** | **5** | **6** | **7** | **8** | **9** | **10** | **11** | **12** | **13** | **14** | **Fund-ing source** | **Quality** | **Applicability(1)** |
| --- | --- | --- | --- | --- | --- | --- | --- | --- | --- | --- | --- | --- | --- | --- | --- | --- | --- |
| Eisenman, 2009(5) and Glik, 2014(6) | Y | Y | Y | Y | Y | Y | Y | Y | Y | Y | N | N | N | N | CDC | Fair | Fair |
| Castillo, 2018(7) | Y | NA | Y | Y | NA | Y | NA | N | Y | Y | Y | NA | Y | N | NR | Good | Fair |

Abbreviations: CDC=Centers for Disease Control and Prevention; N=No; NA=Not applicable; Y=Yes

Criteria (Adapted Newcastle-Ottawa(4)):

Selection

1. Was the exposed cohort representative?
2. Was the non-exposed systematically selected?
3. Ascertainment of exposure reported and valid?
4. Eligibility criteria specified?

Comparability

1. Were the groups comparable at baseline?

Outcome

1. Did the study use accurate methods for ascertaining exposures, potential confounders, and outcomes?
2. Outcome assessors masked?
3. Reporting of attrition, adherence, and contamination?
4. Were outcomes pre-specified and defined, and ascertained using accurate methods?
5. Follow-up long enough for outcomes to occur?
6. Appropriate statistical analyses on potential confounders?
7. Important differential loss to follow-up or overall high loss to follow-up?
8. Appropriate Handling of Missing Data?
9. Evidence of Selective Outcome Reporting?

Table. Quality Ratings for Qualitative Studies

| **Author, Year** | **Section A** | | | | **Section B** | | | **Section C** | **Overall Quality Notes** | **Applicability (1)** |
| --- | --- | --- | --- | --- | --- | --- | --- | --- | --- | --- |
|  | **1** | **2** | **3** | **4** | **5** | **6** | **7** | **How valuable is the research?** |  |  |
| Andrulis, 2011(8) | U | Y | Y | NR | NR | Y | U | Valuable - provides a good framework from which to address barriers in California. | Generally good, lacks some methods reporting. | Fair |
| Aten, 2010(9) | Y | Y | Y | Y | Y | Y | Y | Valuable and likely generalizable to other topics for this community. | No issues. Good qualitative methods described. | Fair |
| Aten, 2011(10) | Y | Y | Y | U | Y | Y | U | Valuable - research resulted in action. | Good quality study, except for snowball sampling. | Fair |
| Wyte-Lake, 2014(11) | U | U | Y | NR | NR | Y | U | Valuable, but suggests more research needed. | Small study, poorly reported | Poor |

Abbreviations: N=No; NR=Not reported; U=Unclear; Y=Yes

Criteria (CASP(12)):

Section A: Are the results valid?

1. Was the research design appropriate to address the aims of the research?
2. Was the recruitment strategy appropriate to the aims of the research?
3. Was the data collected in a way that addressed the research issue?
4. Has the relationship between researcher and participants been adequately considered?

Section B: What are the results?

1. Have ethical issues been taken into consideration?
2. Was the data analysis sufficiently rigorous?
3. Is there a clear statement of findings?

Section C: Will the results help locally?

Appendix Table 1. PICOTS

|  | **KQ1: What factors contribute to disparate infection rates and health-related outcomes among different segments of the population during infectious disease epidemics or pandemics?** | **KQ2: What interventions or intervention components have been used to reduce health inequalities (or identified in preliminary studies) in infectious disease transmission or health outcomes in disasters, infectious disease epidemics or pandemics in the United States?** |
| --- | --- | --- |
| **Populations** | Adult Subgroups: race or ethnicity, socioeconomic status, disability, geographic location (*eg*, urban/rural, high density neighborhoods) | |
| **Intervention/**  **Mediating and Moderating Factors** | Risk of exposure:   - Structural (employment, urban/rural, living arrangement, crowding) - Work-related inability to social distance - Other measures of inability to social distance (childcare access, need for public transport, language or cultural barriers) - Access to clean water and sanitation - Hygiene and health-related behaviors   Susceptibility:   - Comorbid chronic diseases - Immunosuppression - Psychologic and nutritional stress   Access to care:   - Regular health care provider - Insurance - Quality of health care   Discrimination and trust   - Interpersonal mistreatment - Community discrimination - Trust in healthcare systems and government   Information/Knowledge | - Emergency preparedness - Messaging and communication - Employment, telework - Childcare - Health care access |
| **Comparator** | - Comparison group within the same group - Comparison to other groups relevant to the population | - Standard public health response - No intervention or pre-intervention - Other interventions - No comparator necessary for pre-intervention studies |
| **Outcomes** | - Mortality - Health care utilization and access - Infectious-disease-related hospitalizations - Burden of illness - Severity of illness - Loss of job due to epidemic/pandemic/disaster | |
| **Timing** | Related to an infectious disease pandemic or epidemic | Related to an infectious disease pandemic or epidemic, or disaster |
| **Setting** | United States and Territories | |
| **Study design** | Trials, quasi-experimental, observational, descriptive, case series (depending on search yield), qualitative. Systematic reviews will be included if they directly address key questions. If not, reference lists will be pearled. | |

APPENDIX REFERENCES

1. U.S. Preventive Services Task Force. Appendix VII. Criteria for Assessing External Validity (Generalizability) of Individual Studies 2017. Available from: https://www.uspreventiveservicestaskforce.org/Page/Name/appendix-vii-criteria-for-assessing-external-validity-generalizability-of-individual-studies.

2. Price M, Davidson TM, Andrews JO, Ruggiero KJ. Access, use and completion of a brief disaster mental health intervention among Hispanics, African-Americans and Whites affected by Hurricane Ike. Journal of Telemedicine & Telecare. 2013;19(2):70-4. PubMed PMID: 23514936.

3. Wyte-Lake T, Der-Martirosian C, Claver M, Davis D, Dobalian A. Provider Delivery of Emergency Preparedness Education in Home-Based Primary Care. Disaster Medicine & Public Health Preparedness. 2019;13(3):547-54. PubMed PMID: 30378517.

4. Wells GA, Shea B, O'Connell D, Peterson J, Welch V, Losos M, et al. The Newcastle-Ottawa Scale (NOS) for assessing the quality of nonrandomised studies in meta-analyses. Available from: http://www.ohri.ca/programs/clinical_epidemiology/oxford.asp.

5. Eisenman DP, Glik D, Gonzalez L, Maranon R, Zhou Q, Tseng CH, et al. Improving Latino disaster preparedness using social networks. American Journal of Preventive Medicine. 2009;37(6):512-7. PubMed PMID: 19944917.

6. Glik DC, Eisenman DP, Zhou Q, Tseng CH, Asch SM. Using the Precaution Adoption Process model to describe a disaster preparedness intervention among low-income Latinos. Health Education Research. 2014;29(2):272-83. PubMed PMID: 24399266.

7. Castillo EM, Chan TC, Tolia VM, Trumm NA, Powell RA, Brennan JJ, et al. Effect of a Computerized Alert on Emergency Department Hepatitis A Vaccination in Homeless Patients During a Large Regional Outbreak. Journal of Emergency Medicine. 2018;55(6):764-8. PubMed PMID: 30316620.

8. Andrulis DP, Siddiqui NJ, Purtle JP. Integrating racially and ethnically diverse communities into planning for disasters: the California experience. Disaster Medicine & Public Health Preparedness. 2011;5(3):227-34. PubMed PMID: 22003140.

9. Aten JD, Topping S, Denney RM, Bayne TG. Collaborating with African American churches to overcome minority disaster mental health disparities: What mental health professionals can learn from Hurricane Katrina. Professional Psychology: Research and Practice. 2010;41(2):167-73. doi: http://dx.doi.org/10.1037/a0018116.

10. Aten JD, Topping S, Denney RM, Hosey JM. Helping African American clergy and churches address minority disaster mental health disparities: Training needs, model, and example. Psychology of Religion and Spirituality. 2011;3(1):15-23. doi: http://dx.doi.org/10.1037/a0020497.

11. Wyte-Lake T, Claver M, Griffin A, Dobalian A. The role of the home-based provider in disaster preparedness of a vulnerable population. 2014;1.

12. Critical Appraisal Skills Programme. CASP Qualitative Checklist 2018 [27 May 2020]. Available from: https://casp-uk.net/wp-content/uploads/2018/01/CASP-Qualitative-Checklist-2018.pdf.
